# Anticoagulation of HeartMate 3 Left Ventricular Assist Device With Apixaban

**DOI:** 10.1016/j.atssr.2023.09.014

**Published:** 2023-10-02

**Authors:** Emily Cowley, June Chen, Hazal Babadagli, Qiang Fu, Jennifer Conway, Holger Buchholz

**Affiliations:** 1 Department of Pharmacy, Alberta Health Services, Edmonton, Alberta, Canada; 2 Division of Cardiac Surgery, Alberta Health Services, Edmonton, Alberta, Canada; 3 Pediatric Cardiology, Stollery Children's Hospital, Edmonton, Alberta, Canada

## Abstract

We report a case of a 56-year-old man requiring a HeartMate 3 after a myocardial infarction and limited recovery on a temporary ventricular assist device. Because of several gastrointestinal bleeding complications and nonadherence, his antithrombotic therapy was switched from warfarin and aspirin to apixaban 5 mg twice daily. We also determined several peak anti–factor Xa levels for ongoing monitoring. Direct oral anticoagulants may be an option for the prevention of pump thrombosis in patients who are unable to tolerate or refuse warfarin therapy.

Durable left ventricular assist device (LVAD) therapy is an option for patients with advanced heart failure refractory to pharmacologic management. One complication of these devices is the risk of thromboembolic events, including pump thrombosis. The HeartMate 3 (Abbott Cardiovascular) LVAD is associated with lower rates of pump replacement and stroke when it is used with an antithrombotic regimen of aspirin (81-325 mg daily) and warfarin (target international normalized ratio [INR], 2-3).^1^ Whereas the use of direct oral anticoagulants (DOACs) has revolutionized the treatment of stroke prevention in nonvalvular atrial fibrillation and venous thromboembolism, their use in mechanical valves or LVADs is not recommended. We describe the use of an alternative oral anticoagulant in a patient with noncompliance and multiple gastrointestinal (GI) bleeds in conjunction with apixaban anti–factor Xa monitoring.

A 56-year-old man in cardiogenic shock was admitted after a non-ST elevation myocardial infarction. A HeartMate 3 was inserted, and aspirin 81 mg and warfarin (INR target, 2-3) were initiated. Several GI bleeds subsequently developed as a result of gastric and intestinal arteriovenous malformations; manifested as melena, these bleeds were treated with argon plasma coagulation, clips, and Hemospray (Cook Medical). After the second GI bleed, aspirin was stopped. The INR remained labile with a time in therapeutic range of 47.3%, resulting from nonadherence to medications and monitoring.

He now presents with his fourth GI bleed within the first year of LVAD insertion. On admission, the INR was 3.9 and hemoglobin level was 54 g/L. Warfarin was held, vitamin K was given, and 4 units of packed red blood cells were transfused. The following day, the INR decreased to 1.5 and hemoglobin level increased to 98 g/L. He was subsequently transferred to our hospital on day 3. Once the hemoglobin level was stable, an intravenous infusion of heparin was initiated and eventually switched to apixaban 5 mg twice daily.

A peak apixaban anti–factor Xa assay level was ordered at steady state, and the sample was drawn approximately 3.75 hours after the eighth dose. The apixaban concentration was reported as 298 ng/mL by a chromogenic anti–factor Xa activity assay and calibrator within 1 week of collection. Other blood work that day included an INR of 1.8, hemoglobin level of 96 g/L, platelet count of 146 × 10^9^/L, and serum creatinine concentration of 93 μmol/L.

Infectious complications secondary to bacteremia required initiation of rifampin for 6 weeks. Apixaban was switched to enoxaparin to avoid drug-drug interactions, and once rifampin therapy was completed, it was restarted at 5 mg twice daily. Two weeks later, the dose was reduced to 2.5 mg twice daily for bleeding hemorrhoids. A peak apixaban level determined at steady state was 87 ng/mL. We report no significant bleeding or thrombotic events at 6 months.

## Comment

The use of DOACs in LVADs is relatively rare and described in the literature only through case reports or series.^2^, ^3^, ^4^ The largest study to date used apixaban 5 mg twice daily in 47 patients with HeartWare (Medtronic) ventricular assist device in conjunction with either aspirin or clopidogrel.^2^ Our case did not use adjunctive antiplatelets in the hope of minimizing ongoing bleeding.

We elected to initiate a DOAC because of INR monitoring noncompliance and lower rates of major bleeding and similar rates of GI bleeding extrapolated from the atrial fibrillation data.^5^ This is consistent with other case reports documenting noncompliance to warfarin, thromboembolic events, and GI bleeding as reasons for switching.^2^, ^3^, ^4^ Apixaban was selected in our patient because of the superiority to warfarin and the most common agent used in other LVAD patients.^2^ To date, there are no published trials investigating anti–factor Xa levels in LVAD patients. Whereas apixaban levels have not been correlated with clinical outcomes, they are available in-hospital and may offer guidance to evaluate drug exposure.^6^ Routine monitoring of DOAC levels is not common practice and is reserved for urgent clinical situations.^6^ On apixaban 5 mg twice daily, the peak anti–factor Xa level by a chromogenic anti–factor Xa activity assay and calibrator was 298 ng/mL. This level is within the reported ranges in the apixaban monograph (median, 171 ng/mL [91-321]).^7^ Because of bleeding hemorrhoids, the apixaban dose was reduced; a level on 2.5 mg twice daily was 87 ng/mL, which is within the reported range (median, 123 ng/mL; 5th-95th percentile, 69-221).^7^

In conclusion, warfarin remains the anticoagulant of choice for patients with LVADs. However, in a subset of patients who are nonadherent to INR monitoring or in patients with recurrent GI bleeds, a DOAC may be an alternative agent. Our case highlights the potential safety of apixaban with serial anti–factor Xa monitoring in patients with a HeartMate 3 LVAD.
